# Supplementary material for: Content-rich biological network constructed by mining PubMed abstracts
Source: BMC Bioinformatics. 2004 Oct 8;5:147. doi: 10.1186/1471-2105-5-147 (PMC528731; doi:10.1186/1471-2105-5-147)
Supplement: Additional File 5 — The original Chilibot query results of the term "long-term potentiation (LTP)" and 22 other terms, limiting the latest references analyzed to the years 1990, 1995, 2000, and 2004. [file 1471-2105-5-147-S5.bz2 › chilibotAdditionalFile5/ltp2000/html/NMDA_CAMKII.html]

 


 **NMDA** and **CAMKII** 
  
Found 52 abstracts in PubMed,  **30 abstracts were retrieved and analyzed**.  


---

 Search Google  |
 PDF files only 
|  EDU domain only 

---

**Interactive relationship** (e.g. stimulation, inhibition, etc)

- Pretreatment with KN 62, a selective calcium calmodulin dependent protein kinase II  **CaMKII**  inhibitor, abolished the  **NMDA**  action.  Ref: 10792442 Eur J Neurosci, 2000
- **NMDA**  receptors were found to be necessary but insufficient to stimulate  **CamKII** .  Ref: 10375434 Exp Eye Res, 1999
- We have previously shown that autophosphorylation of  **CaMKII**  induces high affinity binding to the NR2B subunit of the  **NMDA**  receptor Strack, S.  Ref: 10764765 J Biol Chem, 2000
- A model in which cooperative interaction between  **NMDA**  and non  **NMDA**  glutamate receptors ion channels is presented to explain the glutamate stimulated increases in  **CamKII**  activity in the retina.  Ref: 10375434 Exp Eye Res, 1999
- Furthermore, mutation of residues in the  **CaMKII**  binding domain in full length NR2B bidirectionally modulates colocalization with  **CaMKII**  after  **NMDA**  receptor activation, suggesting a dynamic model for the translocation of  **CaMKII**  to postsynaptic targets.  Ref: 10764765 J Biol Chem, 2000
- These results suggest that a  **CaMKII**  mediated rise in serine phosphorylation of  **NMDA**  receptor subunits induced by intermittent stimulation of D1 or D2 dopaminergic receptors contributes to the apparent enhancement in striatal  **NMDA**  receptor sensitivity and thus to the dopaminergic response plasticity in levodopa treated parkinsonian rats.  Ref: 10064831 Brain Res, 1999
- The level of antibody binding to  **CaMKII**  alpha, the activity of  **CaMKII** , and the mRNA level for the alpha B subunit of  **CaMKII**  were found to be elevated for short time periods 30 min, 2 h after a single intravitreal injection of  **NMDA** .  Ref: 10762700 Brain Res Mol Brain Res, 2000
- This interaction places  **CaMKII**  not only proximal to a major source of calcium influx but also close to alpha amino 3 hydroxy 5 methylisoxazole 4 propionic acid AMPA type glutamate receptors, which become phosphorylated upon stimulation of  **NMDA**  receptors in these forebrain slices.  Ref: 10077668 Proc Natl Acad Sci U S A, 1999
- Here we show that  **CaMKII**  directly binds to the  **NMDA**  receptor subunits NR1 and NR2B.  Ref: 10077668 Proc Natl Acad Sci U S A, 1999
- Here we report that  **CaMKII**  binds to NR2A and NR2B subunits of  **NMDA**  receptors in PSD isolated from cortex and hippocampus.  Ref: 9751209 J Neurochem, 1998
- It is suggested that regulated expression of  **CaMKII**  alpha B could be involved in the  **NMDA**  mediated cell death in retinal neurons.  Ref: 10762700 Brain Res Mol Brain Res, 2000
- The present results show that calcium entry through  **NMDA**  receptors modulates the Gly receptor function via coactivation of  **CaMKII**  and CaN in the rat SDCN neurons.  Ref: 10792442 Eur J Neurosci, 2000
- Here, we demonstrate that  **CaMKII**  binds to a discrete C terminal region of the NR2A subunit of  **NMDA**  receptors and promotes the phosphorylation of a Ser residue of this  **NMDA**  receptor subunit.  Ref: 10462051 FEBS Lett, 1999
- The association of  **NMDA**  receptor subunits and  **CaMKII**  was assessed by immunoprecipitating PSD proteins with antibodies specific for NR2A B and  **CaMKII**   **CaMKII**  coprecipitated with NR2A B and NR1 but not with other glutamate ionotropic receptor subunits, such as GluR1 and GluR2 3.  Ref: 9751209 J Neurochem, 1998
- **CaMKII** , and calcineurin, resulting in phosphorylation of AMPA receptors by  **CaMKII**  and inactivation of  **NMDA**  receptors at least in part through calcineurin.  Ref: 10218114 Adv Second Messenger Phosphoprotein Res, 1999

**Parallel relationship** (e.g. studied together, co-existance, homology, etc.)

- The  **CaMKII**  dependent phosphorylation of NR2A B subunits of  **NMDA**  receptor is lower in MAM rats when compared to controls 51.77 7.39% of controls level, as revealed in back phosphorylation experiments.  Ref: 9987018 Eur J Neurosci, 1999
- This delivery allowed non functional connections to transmit at resting potentials and required  **NMDA**  receptors  **NMDA**  Rs but not  **CaMKII**  activation.  Ref: 11036266 Nat Neurosci, 2000
- It is shown that N methyl D aspartate  **NMDA**  receptor stimulation reversibly translocates green fluorescent protein tagged  **CaMKII**  from an F actin bound to a PSD bound state.  Ref: 10102820 Science, 1999
- These findings suggest that memory formation of spatial habituation depends on the functional integrity of  **NMDA**  and AMPA kainate receptors and  **CaMKII**  activity in the CA1 region of the hippocampus.  Ref: 11040265 Learn MemLearn Mem, 1999
- Small amounts of the alpha subunit of calcium calmodulin dependent protein kinase II  [ **CAMKII** ]  and subunits of alpha amino 3 hydroxy 5 methyl 4 isoxazolepropionic acid and  **NMDA**  subtypes of glutamate receptors were also detected by immunoblotting.  Ref: 9751204 J Neurochem, 1998
- Calcium influx through the N methyl d aspartate  **NMDA**  type glutamate receptor and activation of calcium calmodulin dependent kinase II  **CaMKII**  are critical events in certain forms of synaptic plasticity.  Ref: 10764765 J Biol Chem, 2000
- To determine whether serine phosphorylation of  **NMDA**  receptor subunits by activation of calcium calmodulin dependent protein kinase II  **CaMKII**  contributes to this process, we examined the effects of unilateral nigrostriatal ablation with 6 hydroxydopamine and subsequent treatment with levodopa, SKF 38393 D1 preferring dopamine agonist, or quinpirole D2 preferring agonist on motor responses and phosphorylation states.  Ref: 10064831 Brain Res, 1999
- These data demonstrate an association between the  **NMDA**  receptor complex and  **CaMKII**  in the postsynaptic compartment, suggesting that this colocalization may be relevant for synaptic plasticity.  Ref: 9751209 J Neurochem, 1998
- In contrast to this, there was a decrease in  **CaMKII**  activity and in the  **CaMKII**  alpha B mRNA levels at longer time periods 24 h following injection of  **NMDA** .  Ref: 10762700 Brain Res Mol Brain Res, 2000
- When the  **NMDA**  channels are open, the determinant role in receptor phosphorylation is played by the PKC and  **CaMKII** .  Ref: 10885014 Ross Fiziol Zh Im I M Sechenova, 2000
- Ten minutes prior to the retention test, through indwelling cannulae placed in the CA1 region of the dorsal hippocampus, they received 0.5 microl infusions of saline, a vehicle 2% dimethylsulfoxide in saline, the glutamate  **NMDA**  receptor blocker, aminophosphonopentanoic acid AP5 5.0 microg, the AMPA kainate receptor blocker, cyanonitroquinoxaline dione CNQX 0.25 or 1.25 microg, the metabotropic receptor antagonist, methylcarboxyphenylglycine MCPG 0.5 or 2.5 microg, the inhibitor of calcium calmodulin dependent protein kinase II  [ **CAMKII** ]  KN62 3.5 microg, the inhibitor of cAMP dependent protein kinase PKA, Rp cAMPs 0.1 or 0.5 microg, the stimulant of the same enzyme, Sp cAMPs 0.1 or 0.5 microg, or the inhibitor of the mitogen activated protein kinase MAPK kinase, PD098059 10 or 50 microM.  Ref: 10840135 Behav Brain Res, 2000
- Usually LTP depends on calcium influx through postsynaptic N methyl D aspartate  **NMDA**  type glutamate receptors and subsequent activation of calcium calmodulin dependent protein kinase II  **CaMKII** .  Ref: 10077668 Proc Natl Acad Sci U S A, 1999
- Dynamic control of  **CaMKII**  translocation and localization in hippocampal neurons by  **NMDA**  receptor stimulation.  Ref: 10102820 Science, 1999
- This study investigated N methyl D aspartate  **NMDA**  mediated cell death and its possible regulation by calcium calmodulin dependent protein kinase II  [ **CAMKII** ]   **CaMKII**  in the adult rat retina.  Ref: 10762700 Brain Res Mol Brain Res, 2000
- The amnesic effect of novelty appears to rely on N methyl D aspartate  **NMDA**  receptor and calcium calmodulin dependent protein kinase II  [ **CAMKII** ]   **CaMKII**  dependent... aspects of its habituation learning.  Ref: 10510197 Eur J Neurosci, 1999
- Novelty causes time dependent retrograde amnesia for one trial avoidance in rats through  **NMDA**  receptor and  **CaMKII**  dependent mechanisms in the hippocampus.  Ref: 10510197 Eur J Neurosci, 1999
- The aims of this study were to investigate the involvement of ionotropic glutamate receptors, especially those of the  **NMDA**  subtype, and the requirement for calcium calmodulin dependent protein kinase II  **CaMKII**  in these phenomena.  Ref: 11114399 Neuropharmacology, 2001
